# A Three-Dimensional DNA Double-Helix Micromixer Enabling Rapid Mixing of High-Viscosity Fluids

**DOI:** 10.3390/mi17101192

**Published:** 2026-10-15

**Authors:** Xin Wang, Yuanlu Fang, Shiliang Song, Zongbo Zhang, Zhanqiang Liu, Luanxia Chen

**Affiliations:** 1 Shandong Key Laboratory of Design and Manufacturing for High-end Offshore Oil and Gas Equipment, China University of Petroleum (East China), Qingdao 266580, China; 2 College of Mechanical and Electronic Engineering, China University of Petroleum (East China), Qingdao 266580, China; 3 School of Mechanical Engineering, Shandong University, Jinan 250061, China; 4 School of Mechanical Engineering, Hefei University of Technology, Hefei 230009, China

**Keywords:** DNA double-helix structure, rotation–revolution flows, chaotic advection, high-viscosity fluids

## Abstract

Rapid and uniform mixing of high-viscosity fluids remains a critical challenge in microfluidics. The inertial effects are strongly suppressed at high viscosity, making it difficult to generate chaotic advection. To address this limitation, a three-dimensional micromixer inspired by the DNA double-helix structure is proposed. The three-dimensional helix microchannel introduces additional torsional forces to generate revolution flows, in addition to the centrifugal forces, which leads to Dean vortices. Fluids not only rotate along the microchannel axis but also revolute along the helix axis. This is analogous to planetary motion, in which a planet spins on its own axis while simultaneously revolving around the sun. Such revolution effects lead to rotation–revolution flows, which significantly enhances chaotic advection. It was found that for 10 cSt silicone oils, the DNA micromixer achieved mixing performance above 98% at *Re* = 40, outperforming the planar serpentine and splitting-and-recombining (SAR) micromixers. Even for higher-viscosity fluids, the DNA micromixer achieved mixing performance above 95% at *Re* = 60 for 50 cSt silicone oil and at *Re* = 120 for 100 cSt silicone oil, whereas that of the traditional passive micromixers fell below 36%. This work demonstrates that the rotation–revolution flows induced by the three-dimensional helix microchannels could effectively intensify chaotic advection, providing an efficient pathway for high-viscosity fluids mixing.

## 1. Introduction

Micromixers are essential components in microfluidic systems, enabling rapid and uniform mixing of fluids at microscale. Owing to the compact size, low reagent consumption, and high sensitivity, micromixers have found widespread applications such as drug screening, biochemical assays, and reaction engineering [1,2,3]. According to the mixing mechanisms, micromixers are commonly classified into active [4] and passive types [5]. Active micromixers utilize external energy fields such as electric [6], acoustic [7], magnetic [8], or thermal fields [9] to perturb the flow fields and enhance mixing. In contrast, passive micromixers rely on geometrical structures to generate secondary flows and chaotic advection without external energy input. Numerous passive designs such as serpentine [10], staggered herringbone [11], baffles [12] and splitting-and-recombining (SAR) structures [13] have been developed to improve mixing performance.

Although a wide range of micromixers have been proposed for the mixing of low-viscosity aqueous fluids, the mixing of high-viscosity fluids remains a critical challenge. For high-viscosity fluids such as human saliva and heavy oils, the large viscous resistance severely suppresses the stretching and folding of fluid elements, resulting in weak mass transfer and poor mixing performance [14,15,16,17]. To overcome these limitations, active strategies have been widely employed to enhance mass transfer at the microscale. Electrokinetic instability and AC electro-osmotic actuation can induce local recirculation to enhance fluid mixing [18,19], while traveling-wave electro-osmotic forcing provides effective control of microfluidic transport [20]. Among various active mixing approaches, acoustically driven bubbles have emerged as one of the most efficient solutions [21,22]. Under acoustic excitation, microbubbles could undergo periodic oscillations and generate intense recirculating flows capable of inducing strong chaotic advection even in highly viscoelastic systems [23].

In addition to the acoustic fields, magnetic fields have also been applied to promote mass transfer within high-viscosity liquids [24]. When actuated by external magnetic fields, the magnetic rotors could rotate to generate vortices in fluids with large viscosity contrasts. The cross-shaped rotors and cross-distributed configurations have been shown to produce the largest vortex regions, thereby dramatically improving mixing performance [25]. It demonstrates that external actuation can effectively enhance high-viscosity fluids mixing. However, despite the excellent mixing capacity, the introduction of external energy fields increases engineering complexity, operational instability and cost. The bulk effects generated in acoustic fields would affect the bio-materials. The manufacture and operation of magnetic rotors are complicated. This dependence limits their scalability toward industrial applications, as well as their integration within portable or low-resource microfluidic platforms.

To avoid the limitations of active micromixers, there have been numerous passive micromixer strategies explored for high-viscosity fluids mixing. Li et al. [26] proposed a micromixer with ω-shaped baffles and mixing chambers to investigate the early folding kinetics of human telomere quadruplex. The asymmetric ω-baffles agitated the flow fields, intensifying tugging and folding of streamlines. This enlarged the interfacial area, reduced the diffusion distance, and consequently accelerated the mixing process. Furthermore, Wu et al. [27] demonstrated that contraction–expansion microchannels can effectively promote mixing of highly viscous fluids through recirculating vortices and interface stretching. Their findings further revealed that concentration-dependent viscosity strongly alters secondary flow distributions.

As the demand for handling high-viscosity fluids has grown, passive micromixers have gradually evolved to more complex three-dimensional structures. Wang et al. [28] designed a three-dimensional splitting-and-recombining (SAR) micromixer, in which fluids were split horizontally and recombined vertically. Such topological reorientation drastically enlarged the interfacial area and generated three-dimensional rotational flows, giving rise to strong chaotic advection. Furthermore, embedding wire or blades was an efficient solution for high-viscosity fluids mixing [29,30]. There has been a helix blade micromixer fabricated via 3D printing to improve the mixing performance of high-viscosity fluids. The helix blade was designed based on the Fibonacci sequence and embedded in the microchannel, which could physically disrupt the laminar flow fields and shorten the diffusion distance [31].

Compared with two-dimensional structures, three-dimensional structures could achieve more efficient mixing. Helical coil-shaped microchannels have been proposed, which demonstrated the important role of three-dimensional channel geometry and hydrodynamics in enhancing convective transport [32]. Moreover, the studies of out-of-plane three-dimensional microchannels showed that increasing the flow rate could induce distinct vortices and substantially modify convective transport [33]. These studies indicated that the sustained three-dimensional chaotic flows and continuous interfacial folding were essential for overcoming diffusion bottlenecks in high-viscosity fluids mixing.

Nevertheless, most passive three-dimensional micromixers designed for high-viscosity fluids require complex curved structures, inserts, or multilayer alignment, which greatly increases the fabrication cost and limits integration with other microfluidic components, thereby restricting their broader adoption. To simultaneously achieve efficient mixing and manufacturability, this study proposes a biomimetic DNA double-helix micromixer. Its structure enables coupled self-rotation and revolution flows in high-viscosity fluids. Unlike complex inserts or multilayer stacks, this three-dimensional structure can be finished via simple assembly. It continuously induces strong interfacial folding and chaotic advection without external field input, offering a novel passive mixing solution for high-viscosity fluids and multiphase systems that is highly efficient, stable, and easily manufacturable.

## 2. Micromixer Design

**Figure 1:**
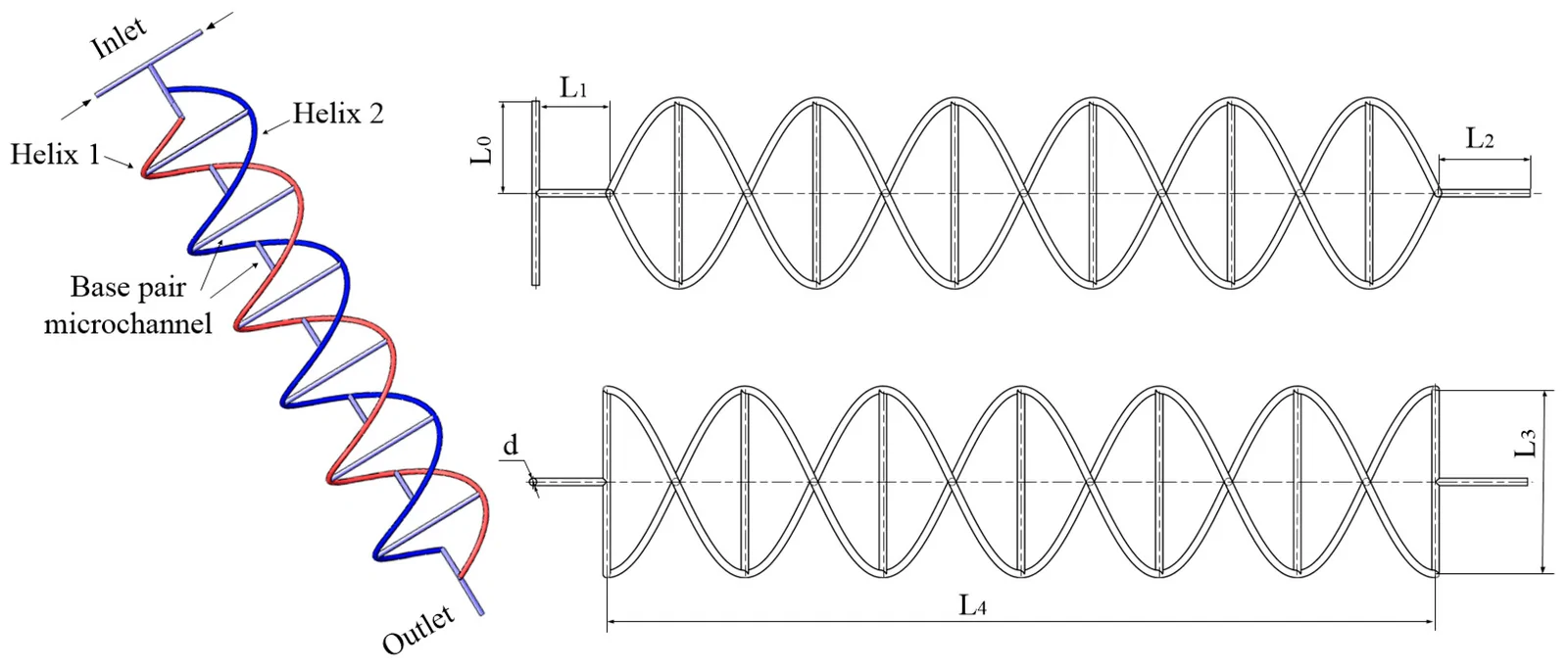
Schematic diagrams of the three-dimensional DNA double-helix micromixer.

Figure 1 presents the schematic diagrams of the three-dimensional DNA micromixer. The DNA double-helix structure is derived from two spatial helix curves. The two helix curves are defined in parametric form, as expressed in Equations (1) and (2), respectively. The phase difference between the two curves is π.

(1) $$r_{1}=(10\mathrm{cost},10\mathrm{sint},\frac{15t}{\pi })$$

(2) $$r_{2}=(-10\mathrm{cost},-10\mathrm{sint},\frac{15t}{\pi })$$

where *r*_1_ and *r*_2_ are the position vectors of helix 1 and helix 2, respectively. *t* is the independent curve parameter. The constant coefficient 10 defines the radius of the circular projection in the *xy*-plane. The factor 15/π determines the vertical pitch of the helix. The height of the helix ranges from 0 to 90 mm. Since the z-coordinate evolves as *z*(*t*) = 15*t*/*π*, the parameter t is constrained as Equation (3).

0 ≤ *z*(*t*) ≤ 90 ⇒ 0 ≤ *t* ≤ 6*π* (3)

Similar to the base pair structure, the two helix curves are connected by multiple interlaced microchannels, as shown in Figure 1. The microchannels have a circular cross-section with a diameter of 0.8 mm. All the detailed parameters of the DNA double-helix micromixer are presented in Table 1.

## 3. Mixing Experiments

**Figure 2:**
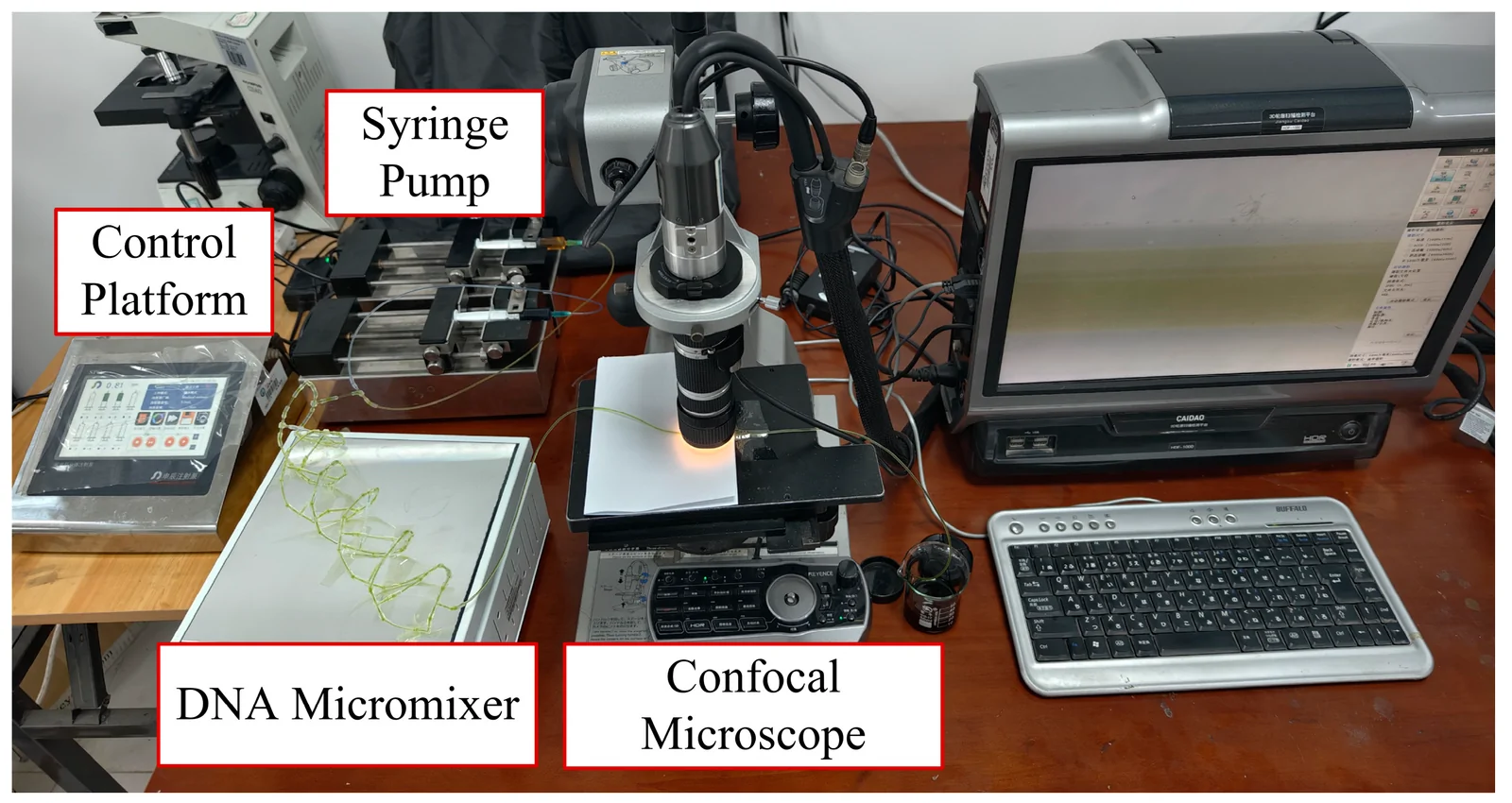
Setup of mixing experiments.

Figure 2 presents the setup of mixing experiments. The three-dimensional DNA double-helix micromixer was manufactured through PTFE tubes, tees and elbow fittings. Compared with the traditional micromixer fabricated by micromilling, soft photolithography and even 3D printing, the three-dimensional DNA micromixer proposed was more convenient to manufacture. Silicone oils with viscosities of 10, 50, and 100 cSt were used as the working fluids. Three drops of oil-soluble blue and yellow dye (approximately 50 μL per drop) were added to 10 mL of silicone oil, solely for flow visualization. Two steady and continuous streams were supplied into the micromixer using syringe pumps.

A confocal microscope was focused at the mid-depth of the outlet microchannel to take snapshots of mixing. The imaging conditions were kept unchanged for all experiments. The outlet images were converted to grayscale using the ImageJ 1.47 software, and the standard deviation of the grayscale intensity was used to evaluate the mixing uniformity. Due to experimental limitations, vertical mixing images were acquired to evaluate mixing performance and validate the numerical simulation. Each experimental condition was independently repeated three times, and the mean value and standard deviation were calculated.

## 4. Numerical Simulation

The flow fields and mixing process of the three-dimensional DNA micromixer have been investigated by numerical simulation, carried out in COMSOL Multiphysics 6.1a software. Assuming that the mixing fluids are incompressible Newtonian fluids, the flow model can be described by the three-dimensional Navier–Stokes equation and continuity equation, expressed in Equations (4) and (5).

(4) $$\nabla \cdot \vec{V}=0$$

(5) $$\rho \left(\vec{V}\cdot \nabla \right)\vec{V}=-P+\mu \nabla^{2}\vec{V}$$

where $\vec{V}$ represents the flow velocity, *μ* is the dynamic viscosity, *ρ* is the fluid density, and *P* denotes the pressure. The concentration distribution in micromixers is modeled according to the convection–diffusion equation, expressed in Equation (6).

(6) $$(\vec{V}\cdot \nabla )c=D\nabla^{2}c$$

where *c* is the concentration. *D* denotes the diffusion coefficient. Rashidnia et al. [34] reported diffusion coefficients of approximately 4.4 ~ 5.4 × 10^−12^ m^2^/s for miscible silicone oil systems, which provided an order-of-magnitude reference. In this research, a representative effective diffusion coefficient of 5 × 10^−12^ m^2^/s is adopted for all three silicone oils as a simplifying assumption. The other fluids characteristics are shown in Table 2.

The Reynolds number (*Re*) that represents the flow conditions is described as Equation (7).

(7) Re=ρVDhμ

where *D_h_* is the hydraulic diameter of microchannels, and *V* is the average flow velocity.

The inlet velocities for different fluids corresponding to Reynolds numbers from 1 to 120 are presented in Table 3.

For the laminar-flow calculation, a P2 + P1 discretization is employed, with quadratic shape functions for the velocity field and linear shape functions for the pressure field. Cubic shape functions are used for the concentration field in the transport of the diluted species interface. No-slip boundary conditions are applied, and the static pressure at the outlet is set to be zero. Two inlets are assigned with fluid concentrations of 1 mol/L and 0, respectively. The uniformity of mixing is evaluated by the parameter mixing performance (*M*), which is calculated based on the standard deviation of the concentration mass fraction (*σ*). The two parameters are defined as Equations (8) and (9).

(8) $$\sigma =\sqrt{\frac{1}{N}\sum_{i=1}^{N}{\left(c_{i}-\bar{c_{m}}\right)}^{2}}$$

(9) $$M=1-\sqrt{\frac{\sigma^{2}}{\sigma_{\max}^{2}}}$$

Here, *σ* denotes the concentration standard deviation. *c_i_* denotes the concentration at each sampling point among the *N* sampling positions at the micromixer outlet. $\bar{c_{m}}$ is the optimal concentration achieved under a completely mixed condition. Since the two inlet streams have equal flow rates and inlet concentrations of 1 mol/L and 0, the completely mixed reference concentration is $\bar{c_{m}}=0.5\;\mathrm{mol}/L$. *σ_max_* represents the standard deviation of a completely unmixed condition. With equal contributions from the two inlet streams, *σ_max_* = 0.5 mol/L. According to Equation (9), an ideal unmixed condition gives *σ* = *σ_max_* and *M* = 0, whereas a completely mixed condition gives *σ* = 0 and *M* = 1.

## 5. Grid Independence Test and Experimental Validation

### 5.1. Grid Independence Test

**Figure 3:**
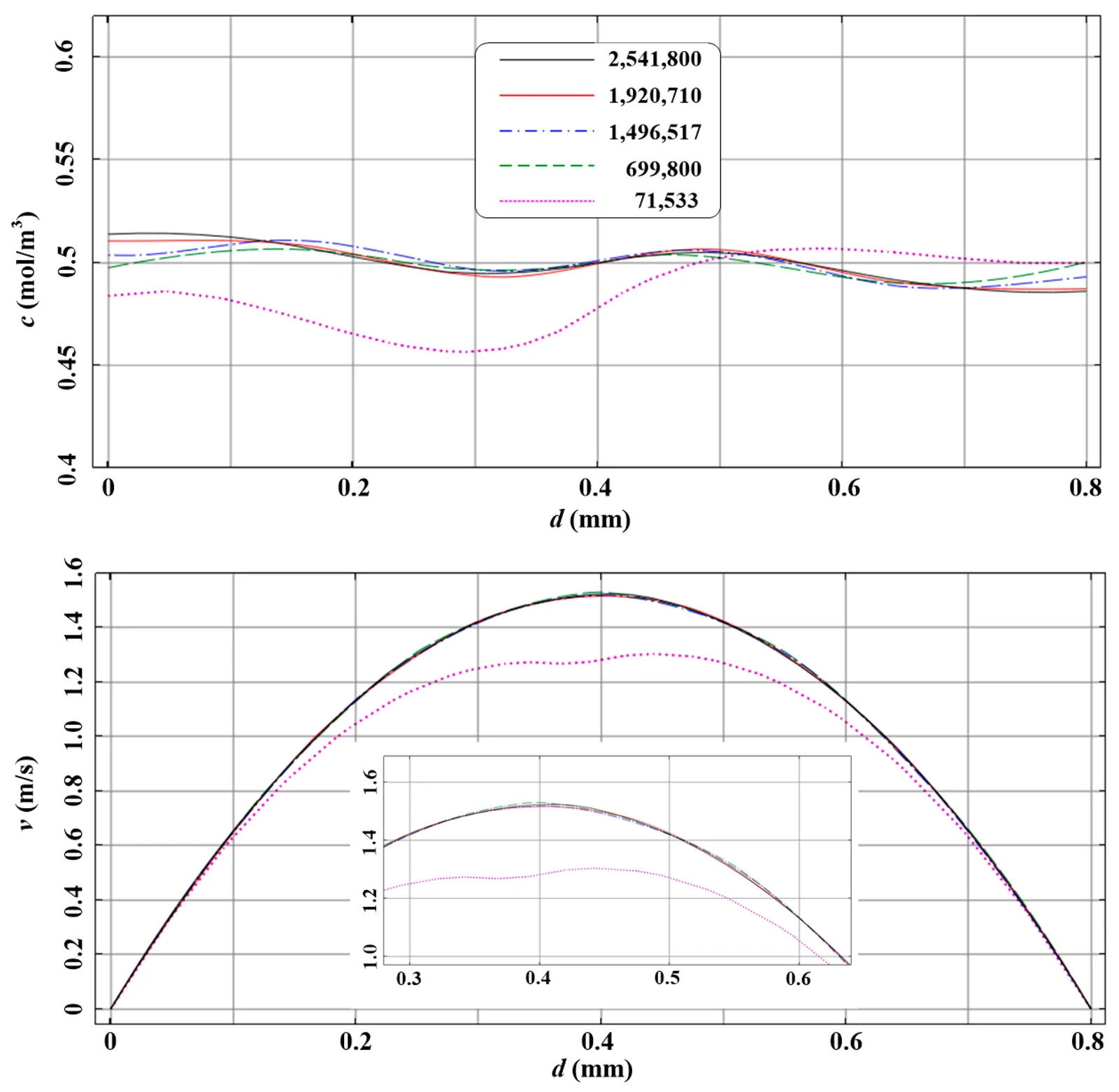
Concentration distribution and velocity distribution of the centerline in outlet at *Re* = 40 for 10 cSt silicone oils.

Considering that the accuracy of numerical simulation is influenced by the mesh partitioning methods, a grid independence test has been conducted to eliminate the impact of mesh discretization on results. Five meshing methods were employed in this study, with grid numbers ranging from 71,533 to 2,541,800. Both the Navier–Stokes equation and the convection–diffusion equation were tested. Figure 3 exhibits the velocity distribution and the concentration distribution of the centerline in the outlet cross-section at *Re* = 40 for the 10 cSt silicone oils.

As presented in Figure 3, it is obvious that beside the meshing method with a grid number of 71,533, the other four meshing methods (with grid numbers from 699,800 to 2,541,800) show a similar velocity distribution and concentration distribution. The velocity distribution of the four meshing methods almost overlaps, while for the concentration distribution, the four meshing methods still show similar results. Especially when the grid numbers are 1,920,710 and 2,541,800, the concentration distribution curves are quite close to each other. The maximum error between the two results is only 0.667%. The corresponding outlet mixing performances were 99.62%, 99.07%, 98.61%, and 98.25%, respectively, further confirming the convergence of mixing performance.

**Figure 4:**
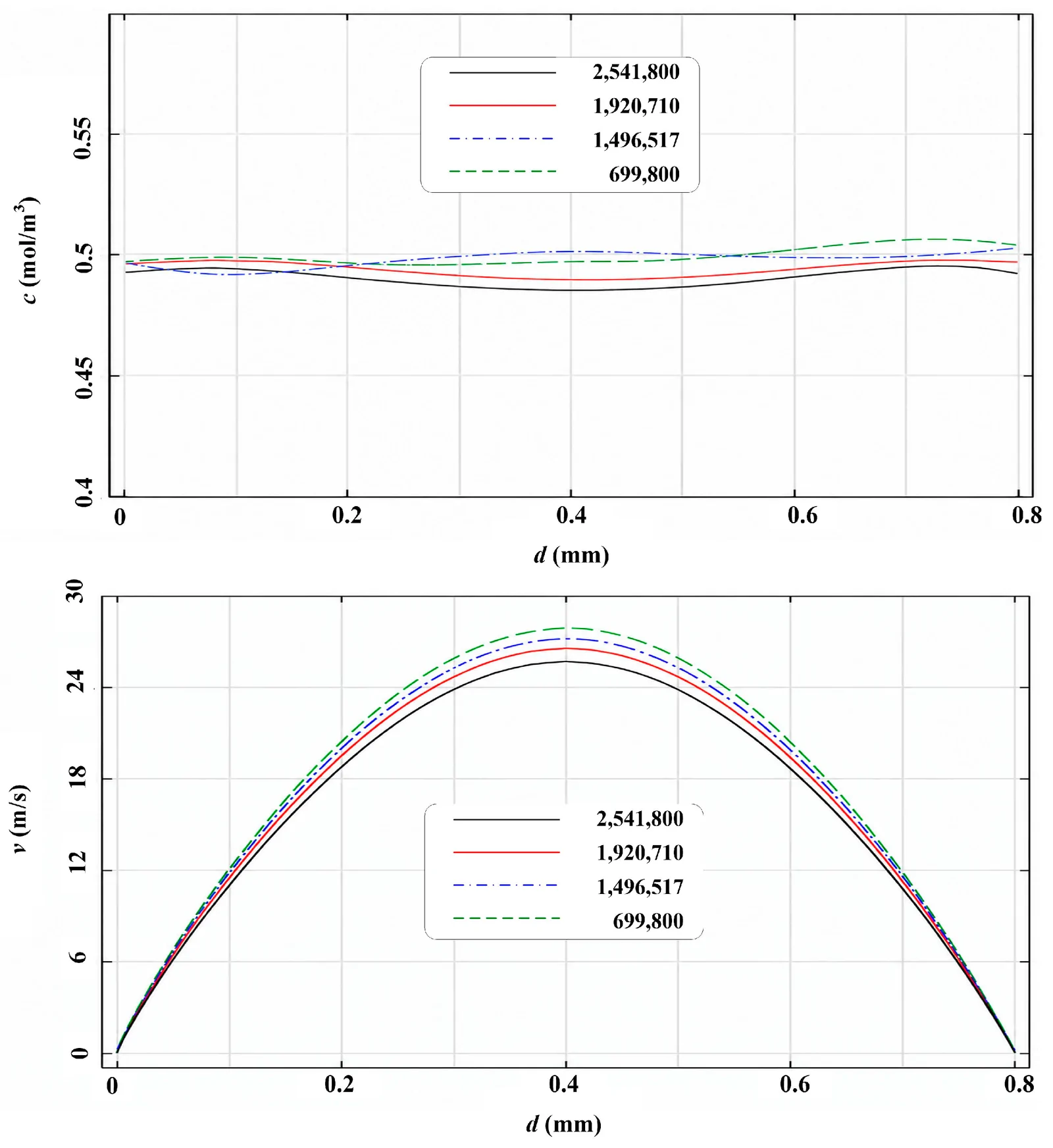
Concentration distribution and velocity distribution of the centerline in outlet at *Re* = 60 for 100 cSt silicone oils.

Moreover, a representative high-viscosity condition was examined to verify the mesh convergence for highly viscous fluids. Figure 4 presents the velocity and concentration distributions along the centerline of the outlet cross-section for 100 cSt silicone oil at *Re* = 60. As shown, the four meshes containing 699,800 to 2,541,800 elements still produced very similar results. In particular, the velocity and concentration profiles obtained with 1,920,710 and 2,541,800 elements nearly overlap, indicating that the numerical results are insensitive to further mesh refinement under the high-viscosity condition. Therefore, considering both numerical accuracy and computational efficiency, the mesh containing 1,920,710 elements was selected for the subsequent simulations.

### 5.2. Experimental Validation

**Figure 5:**
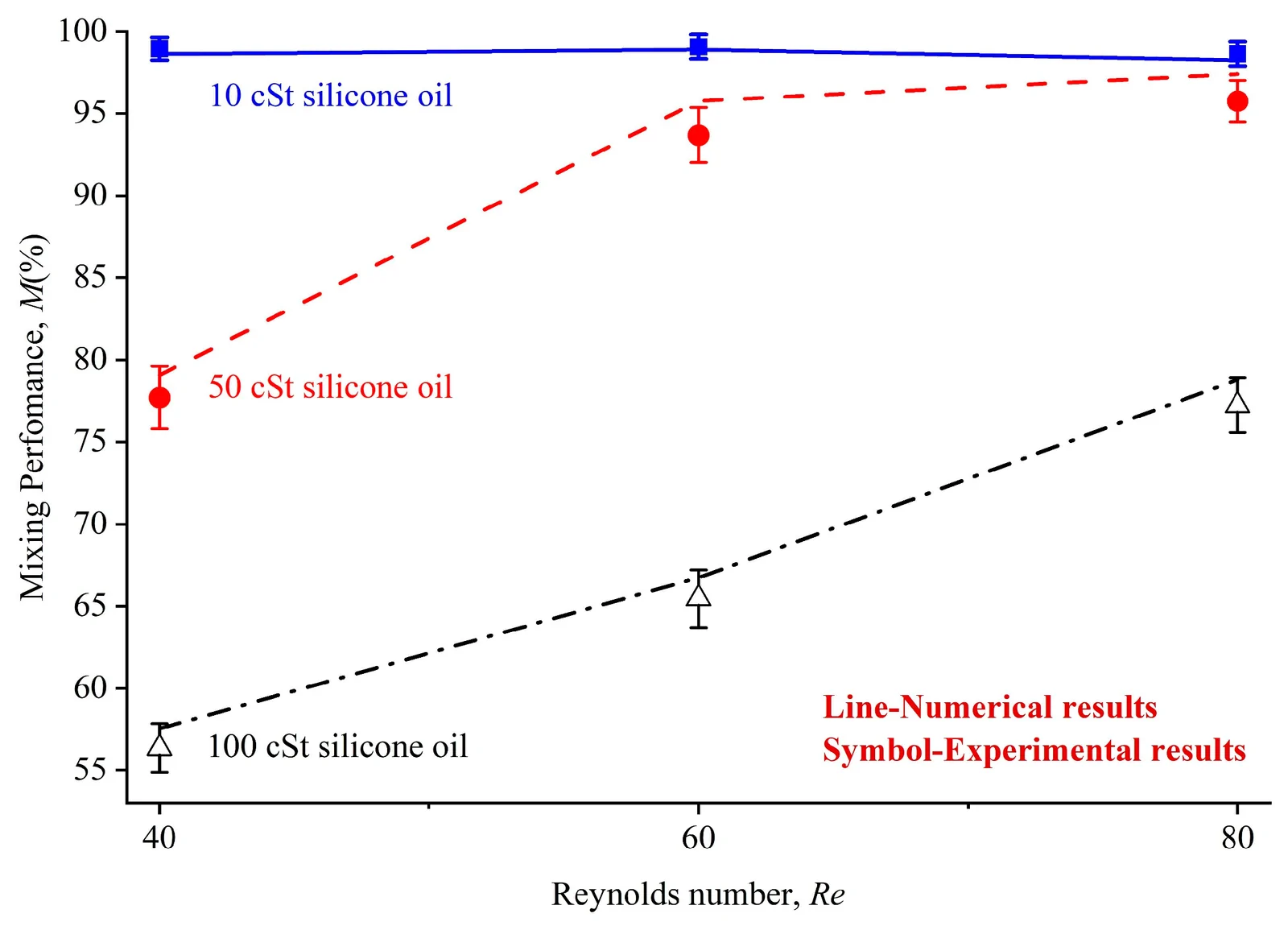
Mixing performance of mixing experiments and numerical simulation.

**Figure 6:**
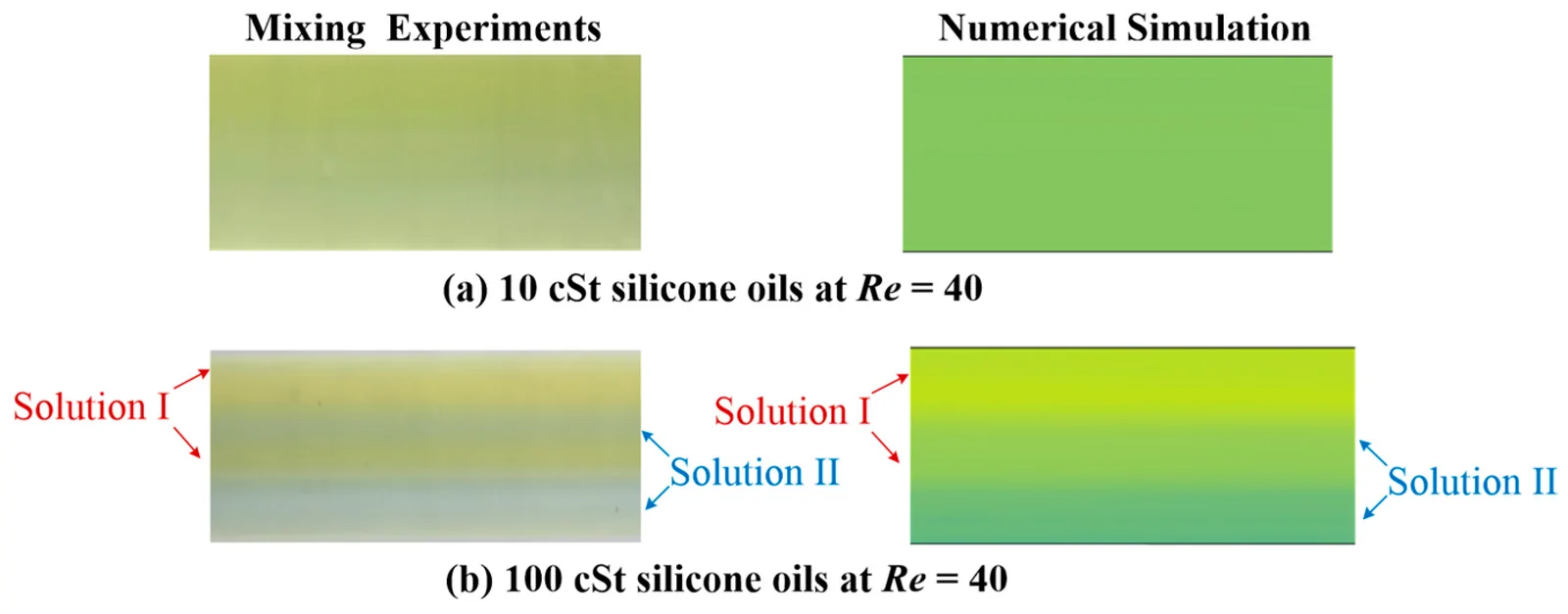
Mixing images of mixing experiments and numerical simulation. (**a**) 10 cSt silicone oils at *Re* = 40; (**b**) 100 cSt silicone oils at *Re* = 40.

**Figure 7:**
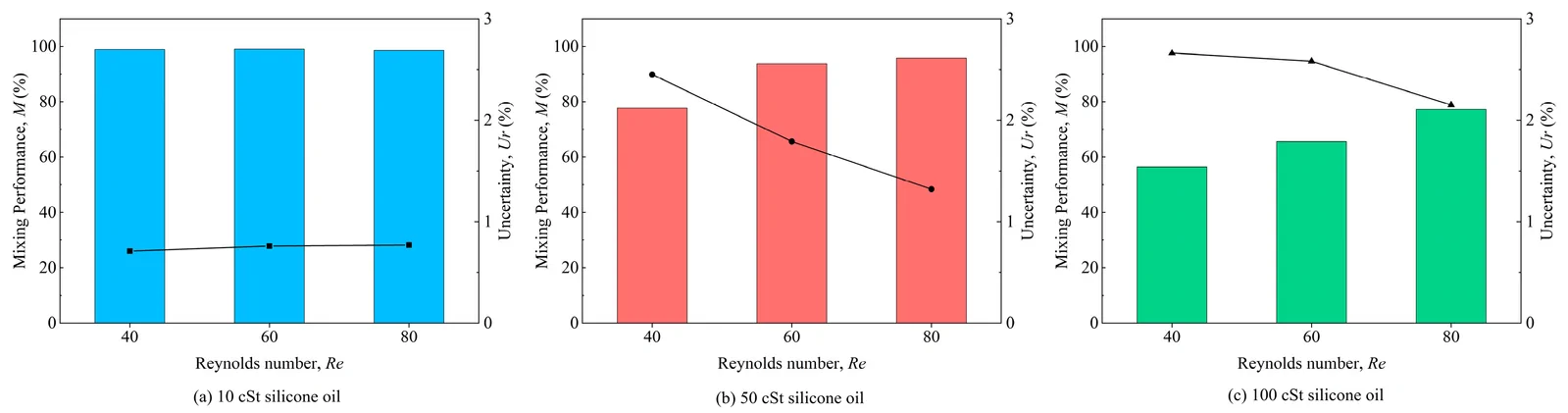
Uncertainty of mixing experiments for (**a**) 10 cSt, (**b**) 50 cSt and (**c**) 100 cSt silicone oils.

The numerical results have been validated by mixing experiments. Figure 5 compares the mixing performance of numerical simulation and mixing experiments. Figure 6 exhibits the mixing images from the top-view direction of numerical simulation and experiments. It can be seen that the numerical results agree well with the experimental results both in mixing performance and mixing images. To further evaluate the repeatability and uncertainty of the experiments, the standard deviation *s* is calculated as Equation (10).

(10) $$s={\left[\sum_{i=1}^{n}\frac{{\left(M_{i}-\bar{M}\right)}^{2}}{n-1}\right]}^{2}$$

where *M_i_* is the mixing performance obtained from each independent experiment, cis the mean value, and *n* is 3 since each experimental condition is independently repeated three times. The relative experimental uncertainty (*Ur*) is evaluated as Equation (11) and presented in Figure 7.

(11) $$\mathrm{Ur}=\frac{s}{\bar{M}}\times 100\%$$

As presented in Figure 7, for the 10, 50, and 100 cSt silicone oils at *Re* = 40, 60, and 80, the relative uncertainties ranged from 0.71% to 2.68%, with a maximum value of approximately 2.66%, indicating good experimental repeatability. As shown in Figure 5, the maximum difference between the experimental and numerical results is approximately 3%, which only occurs for the 50 cSt silicone oils at *Re* = 60 and *Re* = 80. This small discrepancy can be attributed to the different evaluation methods, minor geometric deviations between the fabricated micromixer and the idealized numerical model, and experimental uncertainty. In the numerical simulation, the mixing performance is calculated from the concentration distribution over the outlet cross-section, whereas in the experiments, it is evaluated from the top-view mixing images. In addition, slight deviations in channel geometry and connection transitions may arise during the assembly of the PTFE tubes and fittings, which can locally affect the flow fields and mixing behaviors.

## 6. Results and Discussion

### 6.1. Effects of Reynolds Number on Mixing Process

**Figure 8:**
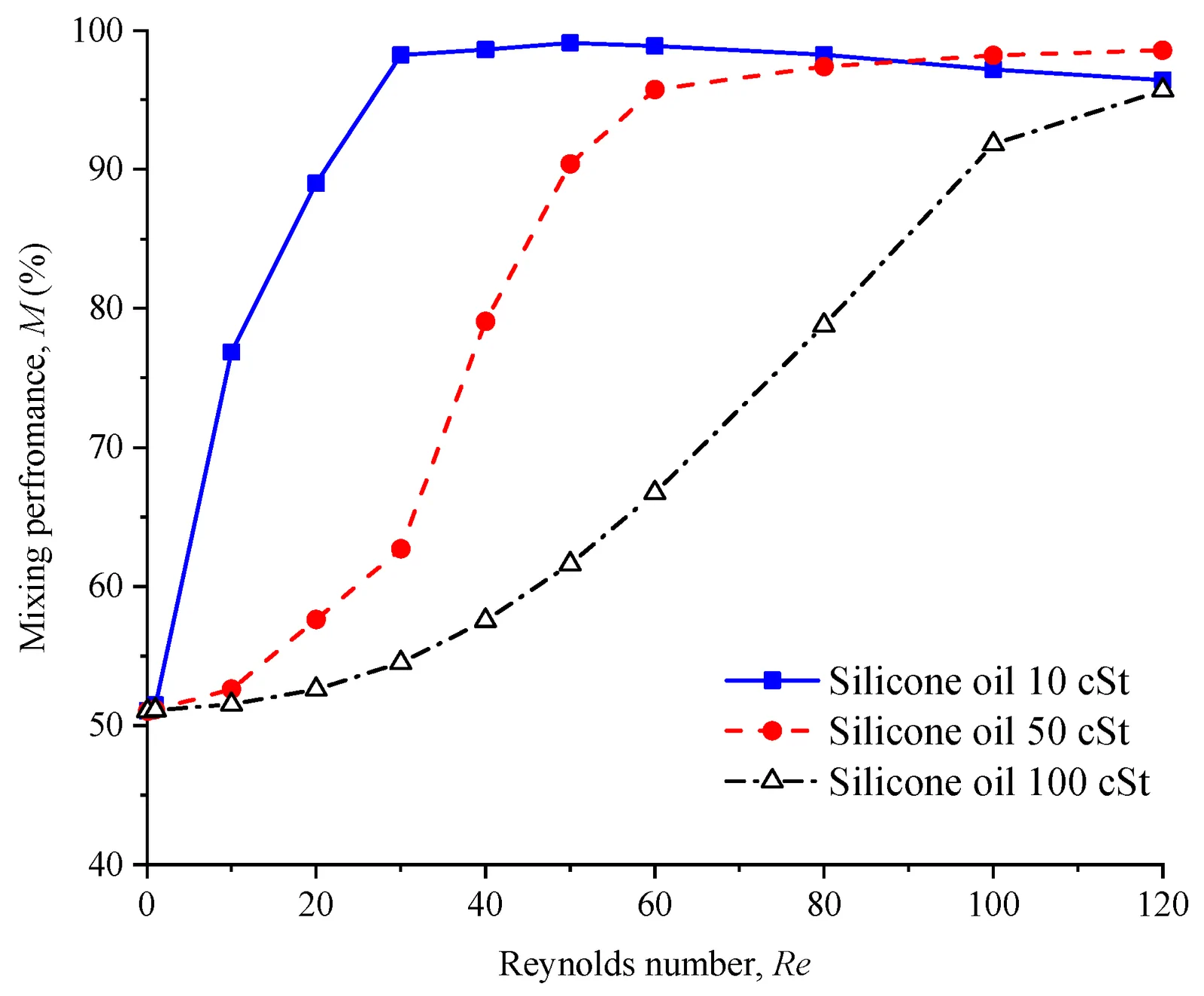
Mixing performance of the DNA double-helix micromixer with *Re* from 0.1 to 120.

Figure 8 exhibits the mixing performance of the three-dimensional DNA double-helix micromixer with *Re* from 0.1 to 120. As presented, for the 10 cSt silicone oil, the mixing performance increases rapidly with the increasing of *Re*. Although the mixing performance is only about 51% at *Re* = 0.1 and *Re* = 1, it rises sharply to exceed 97% once *Re* reaches 30. The most homogeneous mixing occurs at *Re* = 50, with a mixing performance of 99%. When *Re* continues to increase, the mixing performance slightly declines; nevertheless, it remains above 95% even at *Re* = 120.

**Figure 9:**
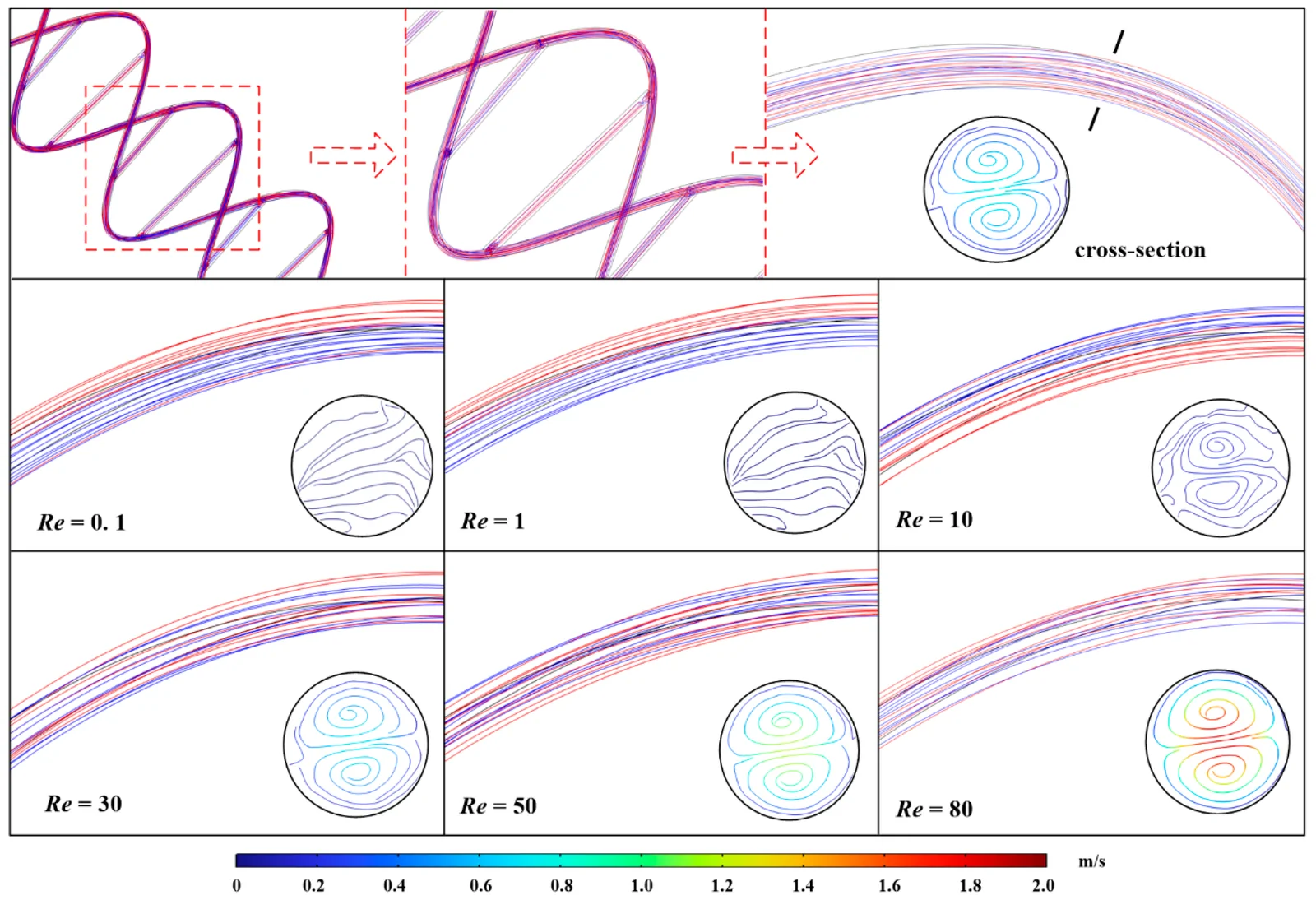
Streamlines of DNA double-helix micromixer for 10 cSt silicone oil.

Figure 9 illustrates the streamlines of the DNA double-helix micromixer for the 10 cSt silicone oil. At low *Re* (*Re* ≤ 1), the streamlines are nearly parallel with each other and exhibit negligible interlacing or perturbation. The flow remains steady and laminar. Under this condition, mixing is primarily dominated by molecular diffusion. The diffusion distance *L_d_* depends on both the residence time and diffusion coefficient, which can be calculated by Equation (12).

(12) $$L_{d}=\sqrt{2Dt_{r}}$$

where *t_r_* is the residence time, and *D* is the diffusion coefficient. Given that the diffusion coefficient of silicone oils typically is on the order of 5 × 10^−12^ m/s, the mixing dominated by molecular diffusion is exceedingly slow. The residence time (*t_r_*) can be calculated by Equation (13).

(13) $$t_{r}=\frac{\left(L_{\mathrm{helix}}+L_{0}+L_{1}+L_{2}\right)}{V}$$

where *L_helix_* is the length of the helix microchannel. According to the definition of Equations (1)–(3), *L_helix_* is 208.88 mm, and the whole flow path is 234.88 mm. *V* is the flow velocity presented in Table 3. The residence time with various *Re* is presented in Table 4.

According to Equation (12), even for a residence time of 1000 s, the diffusion length is only about 100 μm, which is far smaller than the characteristic dimension of a microchannel (800 μm). Therefore, the mixing performance remains nearly constant at around 51% when *Re* ≤ 1. With *Re* increasing to 10, the residence time is less than 2 s for 10 cSt silicone oils, and is even shorter for the 50 and 100 cSt silicone oils, as shown in Table 4. The contribution of molecular diffusion becomes further limited. Meanwhile, as presented in Figure 9, slight distortions in the streamlines begin to appear, which signals the onset of chaotic advection. Chaotic advection enhances mixing by continuously stretching and folding the fluid interface, thereby enlarging the interfacial area and promoting mass transfer. With the further increment of *Re*, the distortion and interlacing between streamlines become more pronounced. Stable and complete Dean vortices are formed at *Re* = 30. The intense chaotic advection leads to rapid and uniform mixing. As shown in Figure 8, for the 10 cSt silicone oil, mixing performance increases rapidly with *Re* increasing from 10 to 50.

At a moderate *Re* (40 ≤ *Re* ≤ 60), the developed Dean vortices bring about strong chaotic advection, resulting in excellent mixing performance exceeding 98%. At higher *Re*, the Dean vortices become more stable and rotationally stronger, as presented in Figure 9. Intuitively, chaotic advection is enhanced, and mixing performance should increase in this condition. However, when *Re* further increases to 80, a slight reduction in mixing performance is observed in Figure 8. This counterintuitive trend can be attributed to vortex entrapment effects.

**Figure 10:**
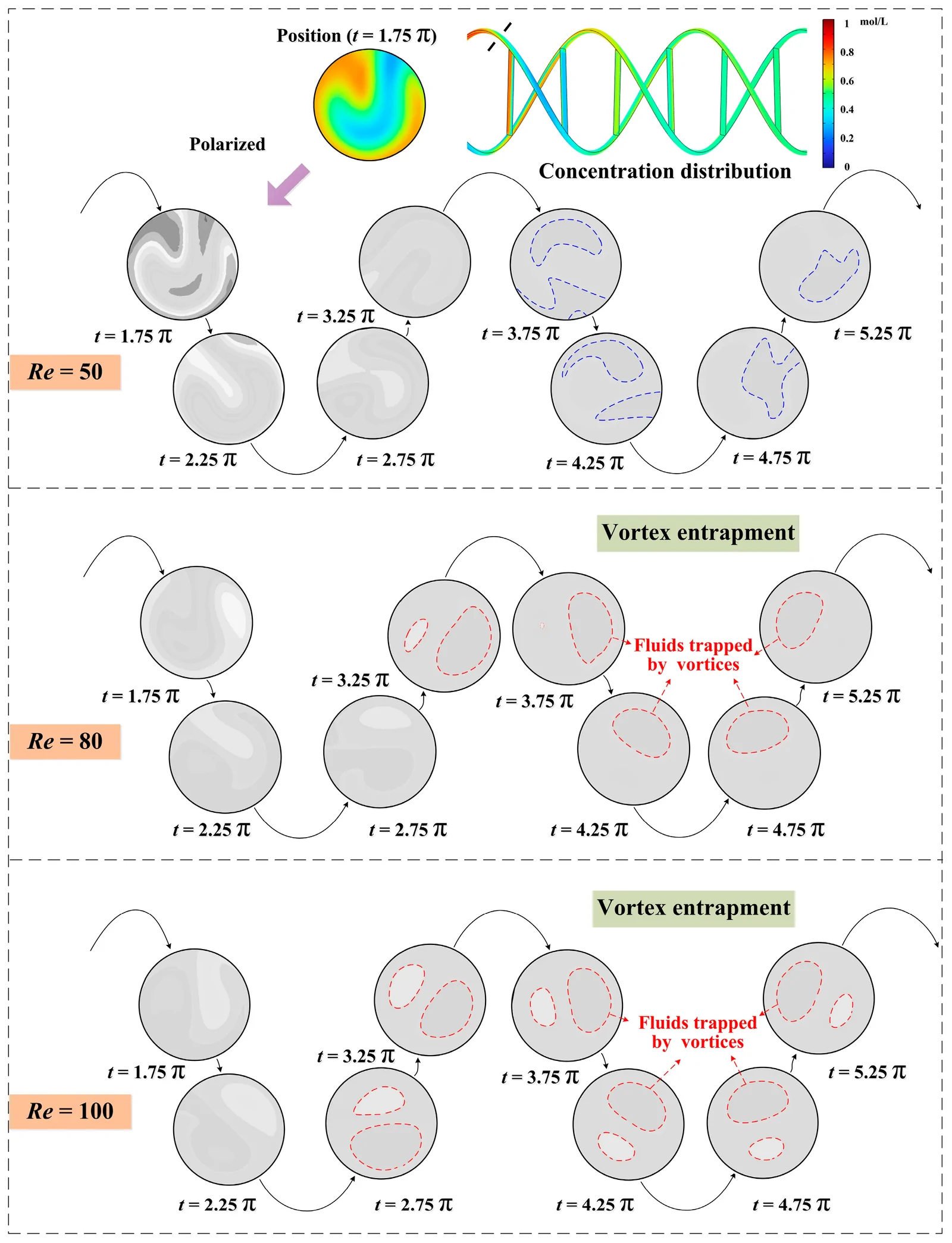
Polarized concentration distribution in cross-sections along the helix microchannel at *Re* = 50 and 100.

Figure 10 illustrates the polarized concentration distribution in cross-sections along the helix microchannel. By polarization, the concentration distribution map can reveal the contour with the greatest concentration gradient. When the contour gradually contracts, the concentration difference is continuously attenuated, indicating progressive homogenization of fluids. In contrast, a nearly unchanged contour suggests limited evolution of the concentration interface and insufficient enhancement of local mass transfer, implying that the mixing process has entered a slowly developing regime.

Compared with the case of *Re* = 50, when *Re* increases to 100, there are two zones where the flow concentration gradient remains higher than the surroundings. The flow near the Dean vortex cores is dominated by the circular motion with very limited radial velocity, which traps the fluids inside the recirculating cells. The mass transfer between the trapped core and the surrounding area becomes very weak. Consequently, there are persistent unmixed zones formed at the center of Dean vortices, which are called vortex entrapment effects.

As shown in Figure 10, the vortex entrapment effects can be faintly observed at *Re* = 80, though they remain weak and there is only one unmixed zone. When *Re* further increases to 100, the vortex entrapment effects become more pronounced. Two distinct unmixed zones take place and expand, accompanied by intensified concentration gradients. It means that there are more fluids trapped in the center of Dean vortices. Although the inertial effects become stronger at high Reynolds numbers, the overly stable Dean vortices hinder the mass transfer. In another word, the excessively high Reynolds numbers can actually lead to a decline in mixing performance. The optimal mixing occurs within a specific range. For the 10 cSt silicone oil, the optimal mixing performance is achieved with *Re* ranging from 40 to 60, where the Dean vortices are sufficiently strong to enhance chaotic advection but not yet stabilized enough to cause the vortex entrapment effects.

### 6.2. Effects of Viscosity on Mixing Process

In addition to the Reynolds number, fluid viscosity plays a pivotal role in influencing flow behavior and mixing performance, especially for oil-phase solutions. Although uniform mixing can be achieved by the three-dimensional DNA double-helix micromixer for the 10 cSt silicone oil, mixing becomes more challenging as the viscosity increases.

As presented in Figure 8, the mixing performance of the 50 cSt silicone oil is only 51% when *Re* ≤ 1, which is comparable to that of the 10 cSt silicone oil. Under the diffusion-dominated mixing process, the effect of viscosity on mixing is negligible since the diffusion coefficient remains constant. However, as *Re* increases, the increment in the mixing performance of the 50 cSt silicone oil proceeds much more slowly compared to that of the 10 cSt silicone oil. Although the residence time of the 50 cSt silicone oil is shorter than that of the 10 cSt silicone oil at the same *Re*, its influence on the overall mixing performance is limited. As shown in Table 4, the residence times are already below 1 s in the moderate *Re* range, and the corresponding molecular diffusion distances are much smaller than the microchannel diameter of 800 μm. Therefore, the difference in mixing performance cannot be mainly attributed to the variation in residence time.

**Figure 11:**
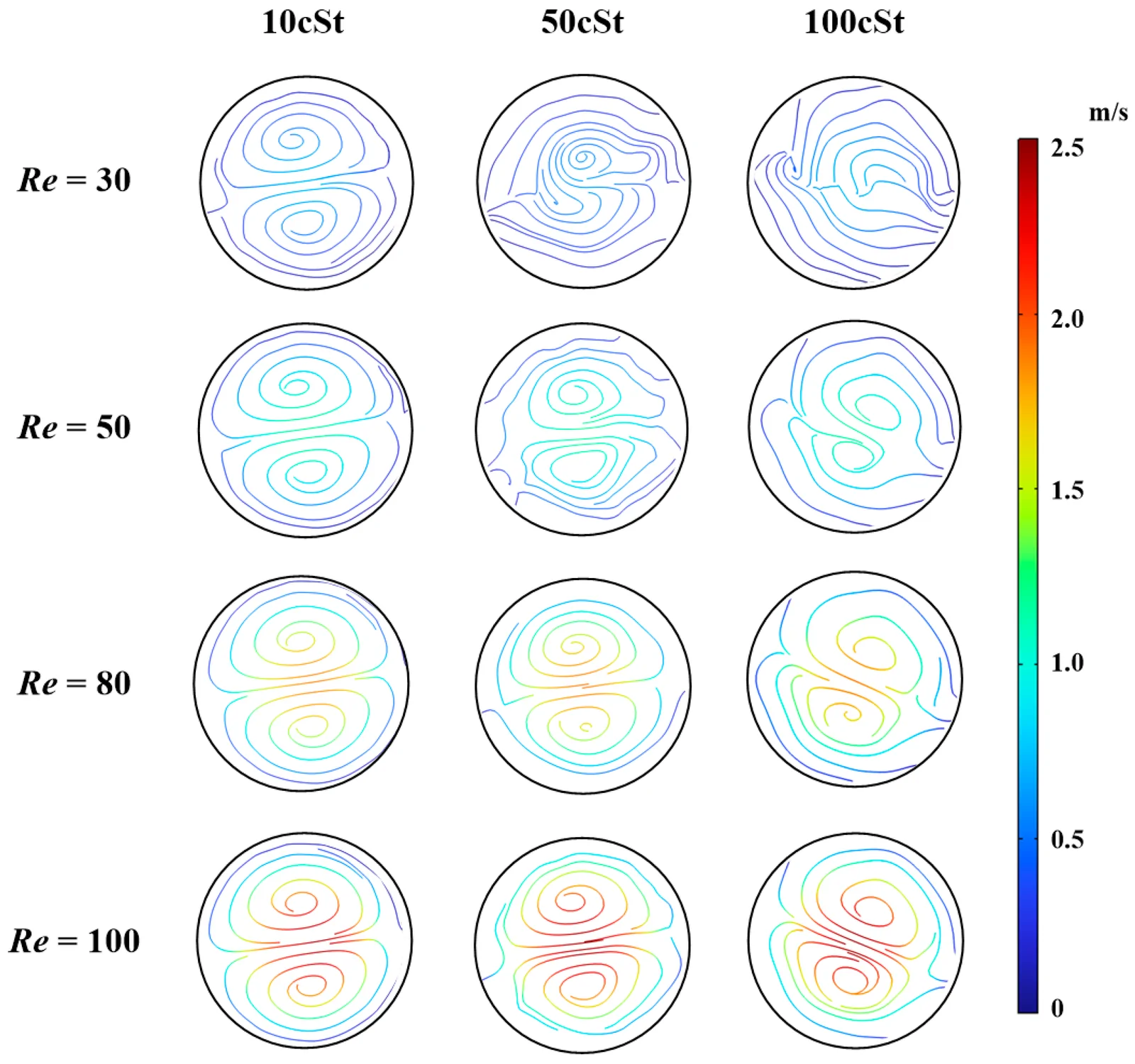
Streamlines of cross-sections of the DNA double-helix micromixer for silicone oil with viscosities of 10, 50 and 100 cSt, respectively.

When *Re* < 30, the mixing performance remains below 60%. Even at *Re* = 40, it reaches 79%, which is still far from complete homogenization. This can be attributed to the elevated viscosity, which enhances the viscous resistance and suppresses the formation of secondary flows. As illustrated in Figure 11, for the 10 cSt silicone oil, complete Dean vortices have already developed at *Re* = 30. In contrast, for the 50 cSt silicone oil, Dean vortices have not yet been formed at the same *Re*. At this stage, chaotic advection remains underdeveloped, and the mixing process is still primarily governed by inefficient molecular diffusion. Only when *Re* increases to 50 are pronounced Dean vortices generated, and the mixing performance approaches 90%.

Furthermore, the mixing of 100 cSt silicone oil is more difficult due to its higher viscosity. As shown in Figure 8, the mixing performance of 100 cSt silicone oil is only 61% at *Re* = 50. Chaotic advection remains weak, and molecular diffusion still dominates. Mixing performance increases slowly with the increase in *Re*. Even at *Re* = 80, the mixing performance remains as low as 78.8%. Owing to the strong viscous damping, it becomes difficult to perturb the steady flow states. Consequently, stronger inertial forces and higher flow velocities are required to sustain effective chaotic advection.

As presented in Figure 11, increasing viscosity substantially alters the morphology of Dean vortices. For the 100 cSt silicone oil, although Dean vortices are formed at *Re* = 80, their shapes deviate from the typical circular patterns and evolve into triangular or wedge-like structures. This is mainly because the strong viscous damping suppresses the radial motion near the microchannel walls, concentrating the residual circulation toward the central region. Dean vortices no longer extend to the microchannel walls but are confined to the central region of the cross-section. Such triangular and wedge-like vortex structure impedes the mass transfer between the central and peripheral flows, resulting in poor mixing performance.

### 6.3. Revolution-Induced Mixing Enhancement

As observed, the DNA double-helix micromixer achieves rapid and uniform mixing of high-viscosity oil-phase solutions under appropriate Reynolds number conditions. This excellent mixing performance arises from the three-dimensional helix microchannel. Within the helix microchannel, the fluids are subjected not only to the centrifugal forces arising from the curvature of the flow path but also to the torsional forces induced by the axial rotation of the helix. Consequently, the three-dimensional helix microchannels generate stronger chaotic advection, compared with the two-dimensional serpentine microchannels or spiral microchannels. To further characterize the flow behaviors in the helix microchannels, the Q-criterion is employed to identify the evolution of vortices, which is defined as Equations (14)–(17).

(14) $$Q=\frac{{\left(\left\|\Omega \right\|-\left\|S\right\|\right)}^{2}}{2}$$

(15) $$\Omega =\frac{\left(\nabla u-\nabla u^{T}\right)}{2}$$

(16) $$S=\frac{\left(\nabla u+\nabla u^{T}\right)}{2}$$

**Figure 12:**
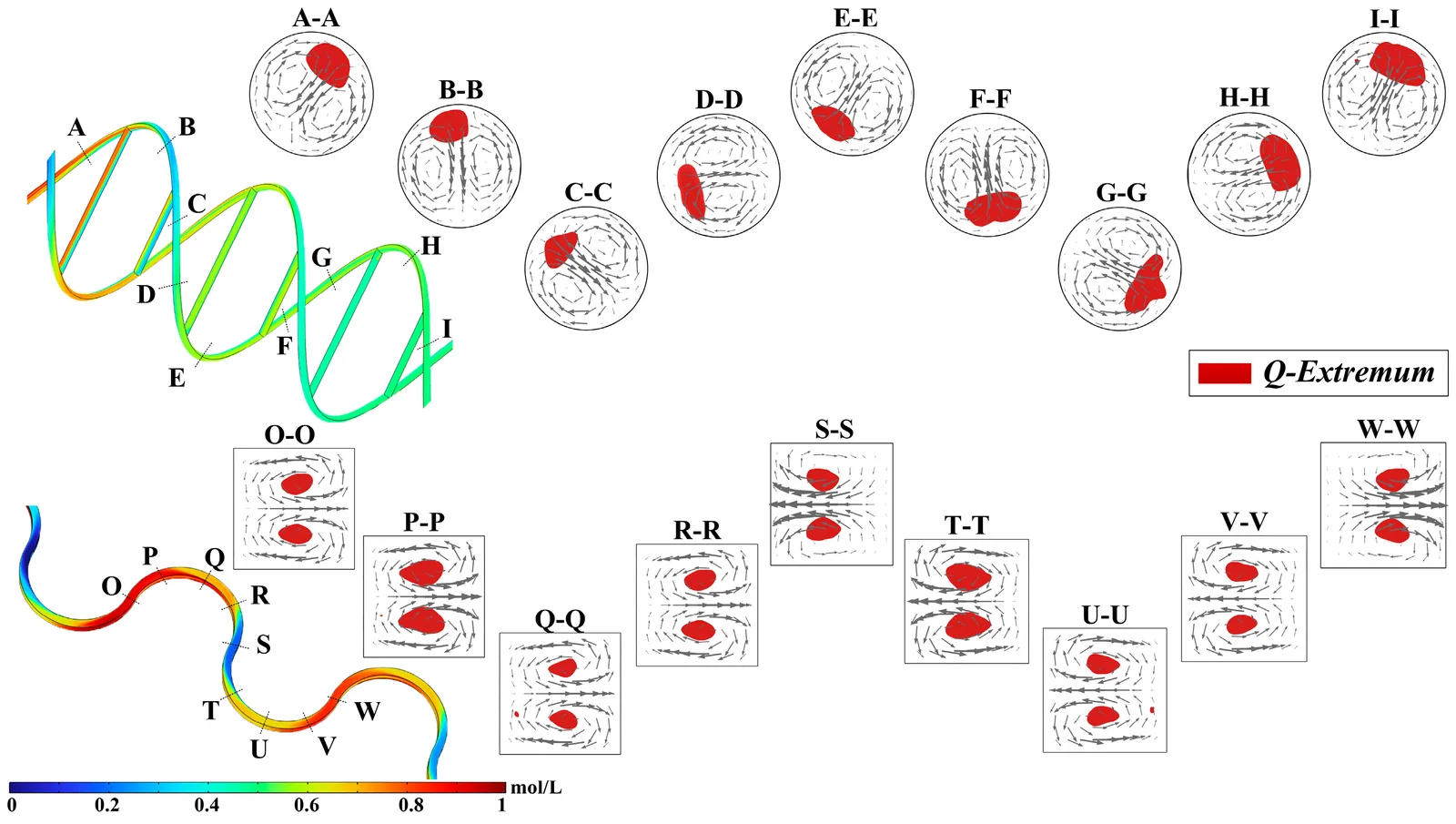
Extremum of *Q* and velocity vectors in cross-sections along the helix microchannel and serpentine microchannel at *Re* = 50.

**u** = (*u*,*v*,*w*) (17)

where *u*, *v* and *w* represent the flow velocity in the *x*, *y* and *z* directions, respectively. Ω and *S* denote the antisymmetric (vorticity) and symmetric (strain rate) components of the velocity gradient tensor, respectively. Regions with *Q* > 0 indicate that the local rotation rates dominate over the strain rates. Thus, the extremum value of *Q* reflects the region dominated by rotational effects and typically corresponds to the vortex cores. Figure 12 exhibits the *Q-extremum* and velocity vectors in cross-sections along the three-dimensional helix microchannel at *Re* = 50. Also, the *Q-extremum* and velocity vectors in a two-dimensional serpentine microchannel are also exhibited for comparison.

As presented in Figure 12, the *Q-extremum* rotates clockwise along the three-dimensional helix microchannel. It suggests a continuous evolution of the vortex core with axial progression. While in the two-dimensional serpentine microchannel, the *Q-extremum* remains fixed at the two centers of Dean vortices. This is because the serpentine microchannel possesses only planar curvature without torsional deformation. The secondary flows are solely induced by the radial centrifugal forces. As a result, the Dean vortices maintain up and down symmetry, and the vortex cores remain stationary throughout the flow. In contrast, the three-dimensional helix microchannel introduces both curvature *κ* and torsion *τ*, which describe the bending and twisting characteristics of the helix structure. A regular helix features constant curvature and torsion, which can be analytically calculated as Equations (18) and (19).

(18) $$\kappa =\frac{R}{R^{2}+C^{2}}$$

(19) $$\tau =\frac{C}{R^{2}+C^{2}}$$

By substituting the geometric parameters of the helix used in this research (*R* = 10, *C* = π/15), the curvature *κ* and torsion *τ* are calculated as 0.08145 and 0.03888, respectively. Owing to the combining effects of curvature and torsion, the *Q-extremum* rotates along the helix microchannel, as presented in Figure 10. Unlike the serpentine microchannel where Dean vortices remain fixed, vortices in the helix microchannel exhibit both self-rotation (Dean vortices) and revolution (*Q-extremum* rotation) around the helix axis. This behavior is analogous to planetary motion, in which a planet spins on its own axis while simultaneously revolving around the sun. Such revolution effects significantly enhance chaotic advection, leading to the rapid and uniform mixing.

### 6.4. Pressure Drop and Mixing Cost

**Figure 13:**
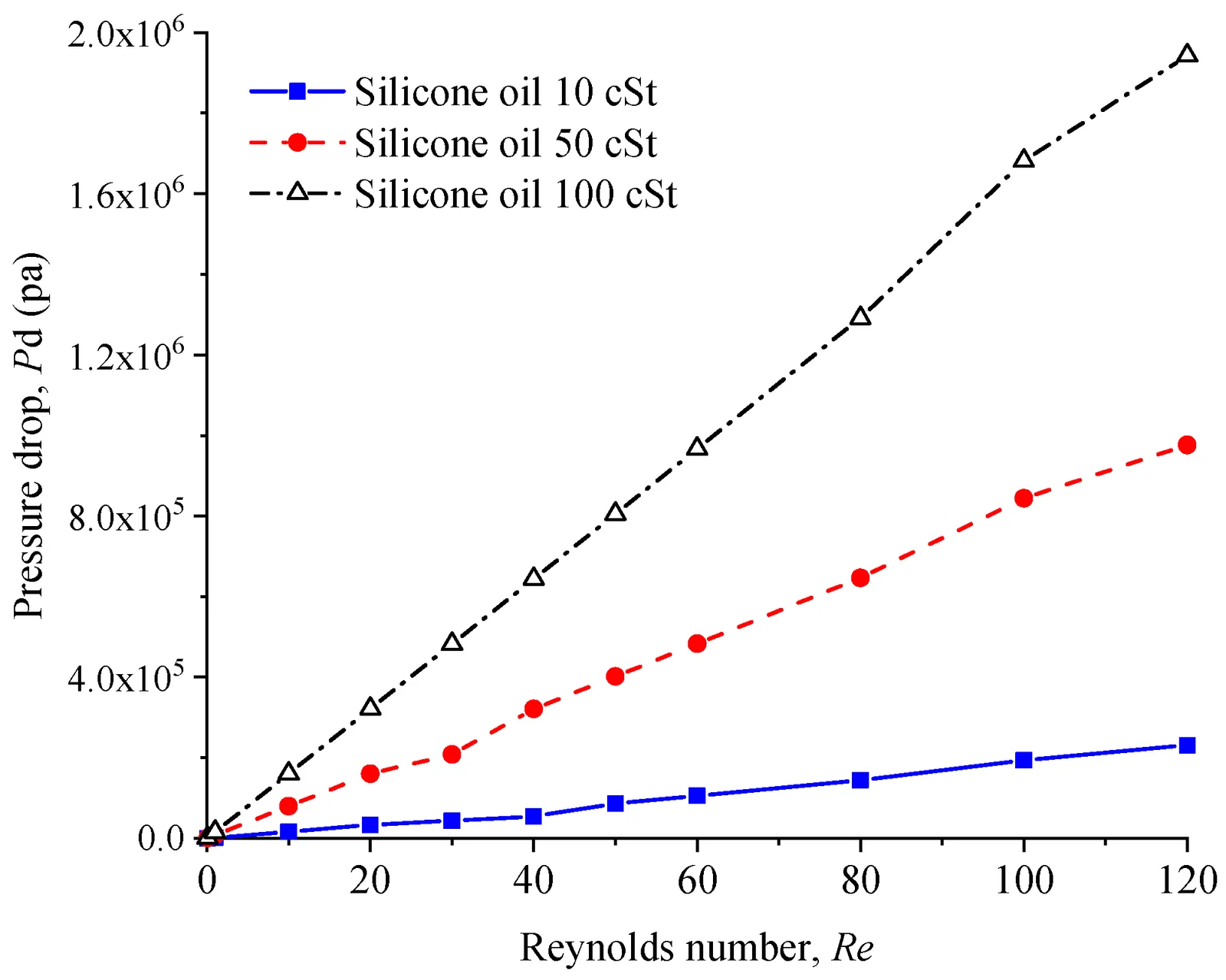
Pressure drop of the DNA double-helix micromixer for silicone oils with *Re* from 0.1 to 120.

Besides mixing performance, the pressure drop (*pd*) is another crucial parameter to be considered in microfluidic systems. As shown in Figure 13, for oil-phase solutions at the same *Re*, those with higher viscosity exhibit higher *pd*, because the higher viscosity results in greater flow resistance in the microchannel. This also reflects the intrinsic challenge of mixing oil-phase fluids with high viscosity. Moreover, *pd* increases with the increase in *Re* since the increased flow velocity intensifies the viscous dissipation and frictional losses along the microchannel. Therefore, an appropriate range of *Re* should be carefully selected to achieve a balance between efficient mixing and acceptable pressure drop.

**Figure 14:**
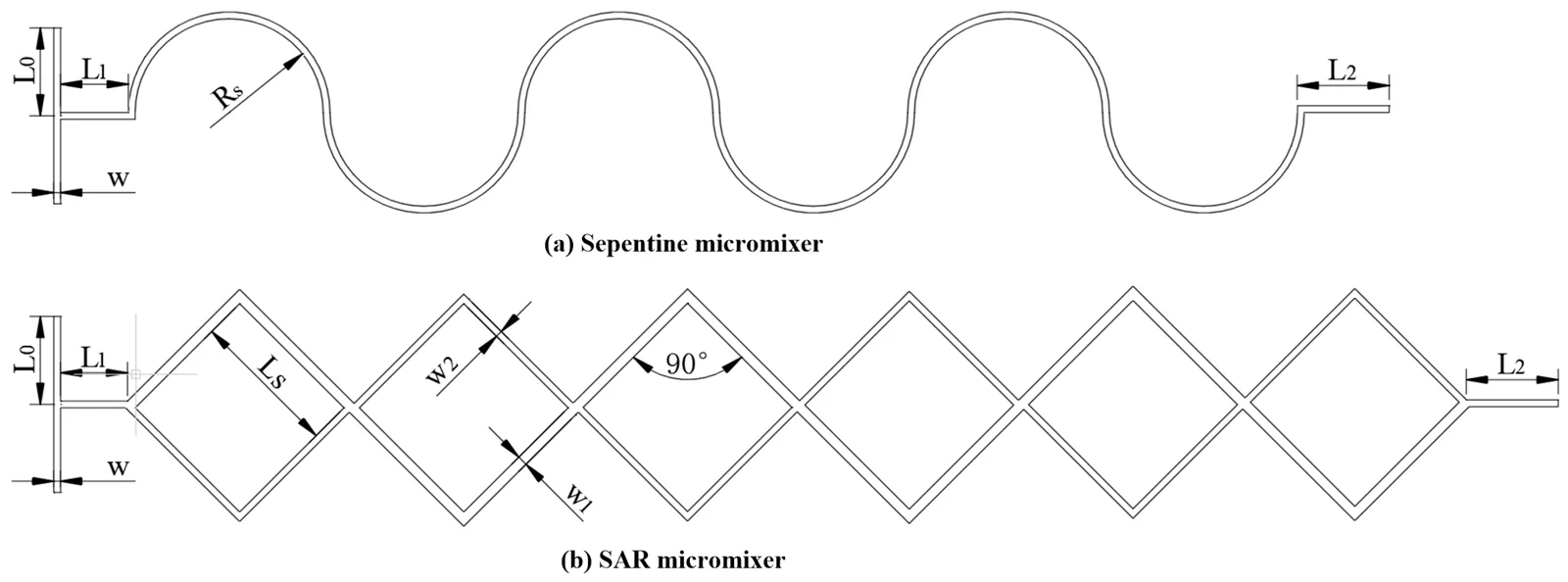
Schematic diagrams of the two-dimensional (**a**) serpentine micromixer and (**b**) SAR micromixer.

For comparison study, the mixing performance and pressure drop of the serpentine and the splitting-and-recombining (SAR) micromixers have also been investigated. Figure 14 exhibits the schematic diagrams of the two micromixers. The characteristic dimensions of these two micromixers are identical to those of the DNA double-helix micromixer for a fair comparison, as shown in Table 5. The inlet length (L_0_), straight channel length (L_1_), outlet length (L_2_) and whole flow path length are identical to those of the DNA micromixer. The channel depth (d_s_) is equal to the channel width (w).

**Figure 15:**
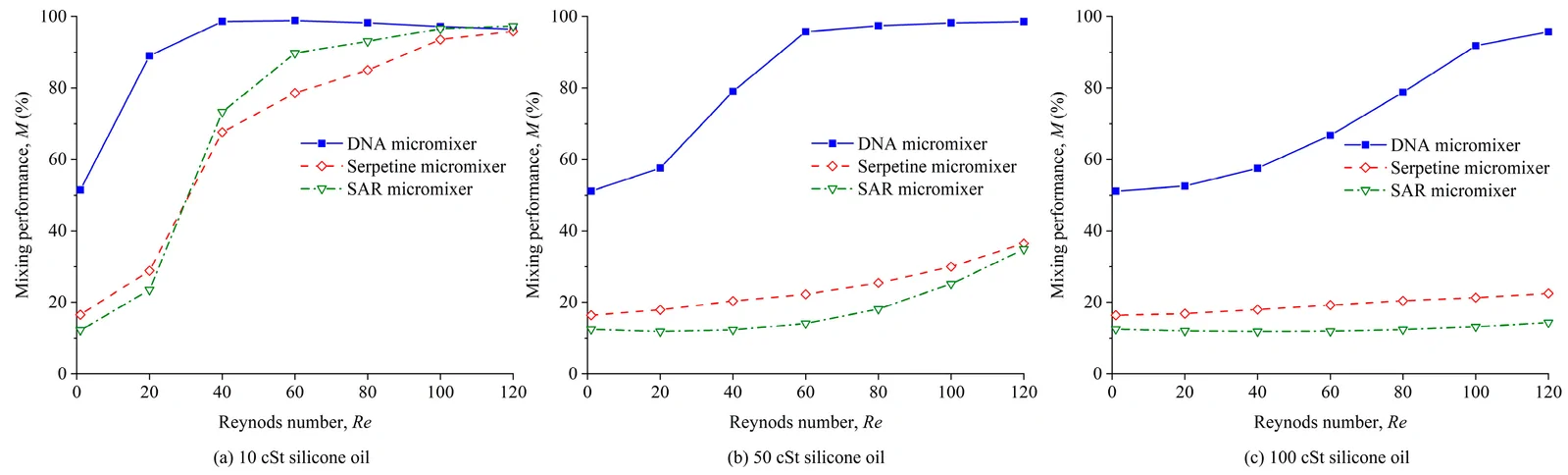
Mixing performance of DNA double-helix micromixer, serpentine micromixer and SAR micromixer for (**a**) 10 cSt, (**b**) 50 cSt and (**c**) 100 cSt silicone oils.

**Figure 16:**
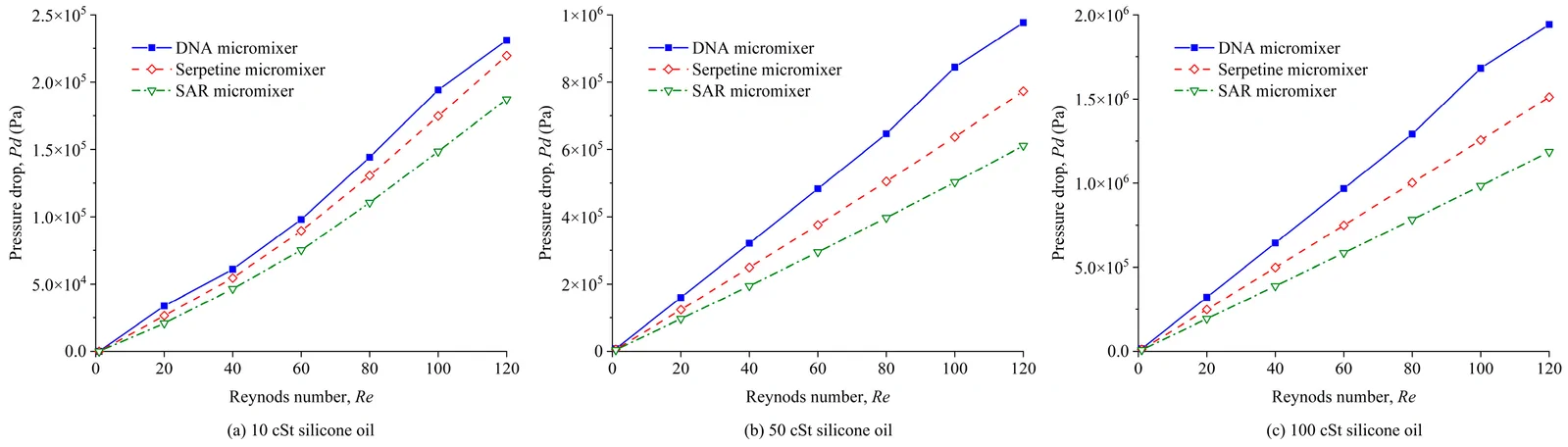
Pressure drop of DNA double-helix micromixer, serpentine micromixer and SAR micromixer for (**a**) 10 cSt, (**b**) 50 cSt and (**c**) 100 cSt silicone oils.

For the 10 cSt silicone oil, both the serpentine and SAR micromixers can achieve high mixing performance, but only at relatively high Reynolds numbers, as shown in Figure 15a. The increase in *Re* strengthens chaotic advection to promote fluids mixing, but also leads to a substantial increase in pressure drop, as shown in Figure 16a. In contrast, the DNA double-helix micromixer achieves a mixing performance of approximately 99% at *Re* = 40, with a corresponding pressure drop of only 54,578 Pa. This indicates that the DNA micromixer can achieve highly efficient mixing with substantially lower pressure loss.

The advantage of the DNA micromixer becomes more pronounced for higher-viscosity fluids. As presented in Figure 15b,c, for the 50 cSt and 100 cSt silicone oils, the maximum mixing performances of the serpentine micromixer are only 36% and 22%, respectively. The SAR micromixer exhibits even lower mixing performance under the same conditions. It suggests that the traditional two-dimensional micromixers fail to generate sufficient chaotic advection for highly viscosity fluids. In contrast, attributed to the revolution-induced mixing enhancement, the DNA double-helix micromixer achieves efficient and uniform mixing even for highly viscosity fluids. The mixing performance surpasses 95% at *Re* = 60 for 50 cSt silicone oil and similarly exceeds 95% at *Re* = 120 for 100 cSt silicone oil, demonstrating its remarkable adaptability and superior performance in handling highly viscous fluids.

**Figure 17:**
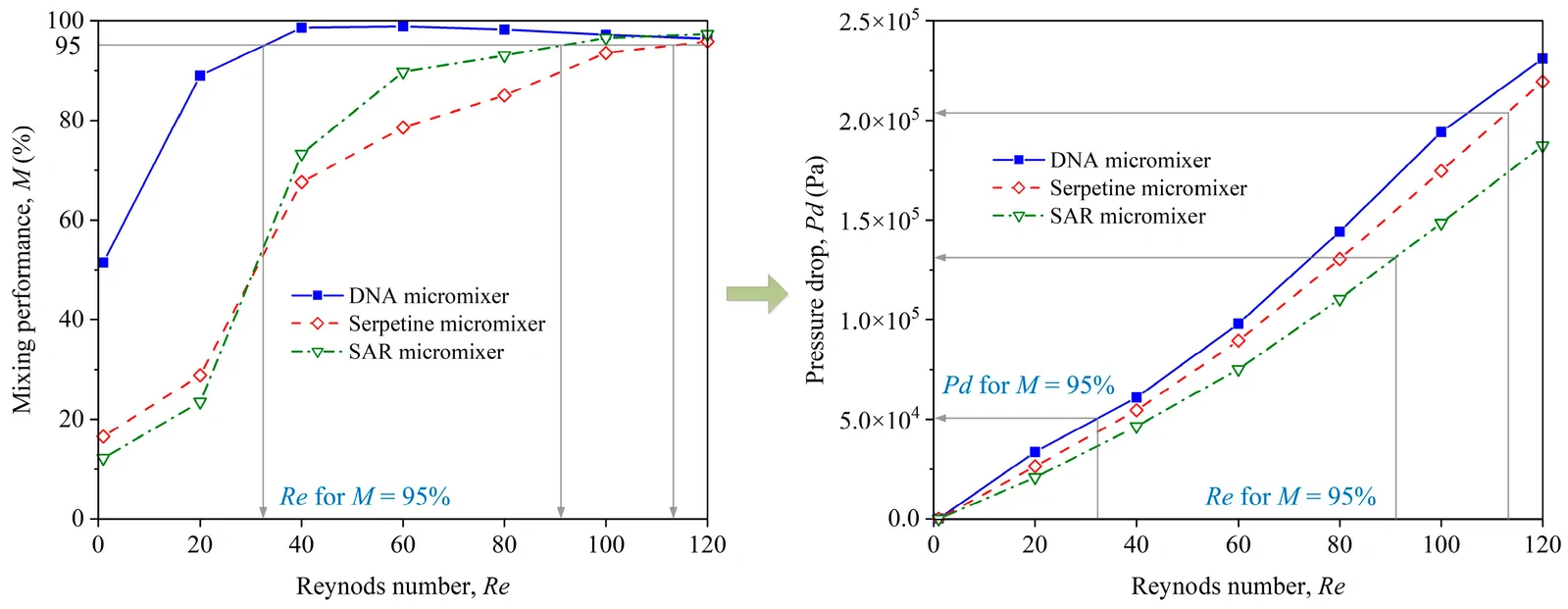
Determination of the pressure drop required to achieve 95% mixing performance for different micromixers.

To quantitatively compare the pressure loss required for achieving the same mixing target, *M* = 95% is selected as the reference, as illustrated in Figure 17. Firstly, a horizontal line corresponding to *M* = 95% is used to determine the Reynolds number required by each micromixer to reach this target. Then, the obtained *Re* is transferred to the corresponding pressure drop curves, and the pressure drop at each *Re* is read directly from the pressure drop plot. Therefore, the pressure drop required to achieve *M* = 95% could be obtained for each micromixer.

As presented in Figure 17, for the 10 cSt silicone oil, the Reynolds numbers required for the DNA micromixer to achieve *M* = 95% is between 30 and 35. In comparison, the Reynolds numbers required for the serpentine and SAR micromixers are above 90 and 110, respectively. The corresponding pressure drops are approximately 5 × 10^4^ Pa, 2.0 × 10^5^ Pa, and 1.3 × 10^5^ Pa, respectively. Notably, the pressure drop required by the DNA micromixer to achieve *M* = 95% is substantially lower than those of the serpentine and SAR micromixers. For the 50 and 100 cSt silicone oils, the DNA micromixer can still achieve a mixing performance above 95% at *Re* = 60 and *Re* = 120, respectively, whereas the maximum mixing performance of the serpentine and SAR micromixers remains below 40%. These results demonstrate that the DNA double-helix micromixer can achieve high mixing performance with a lower pressure drop requirement while maintaining superior mixing capability for high-viscosity fluids.

**Figure 18:**
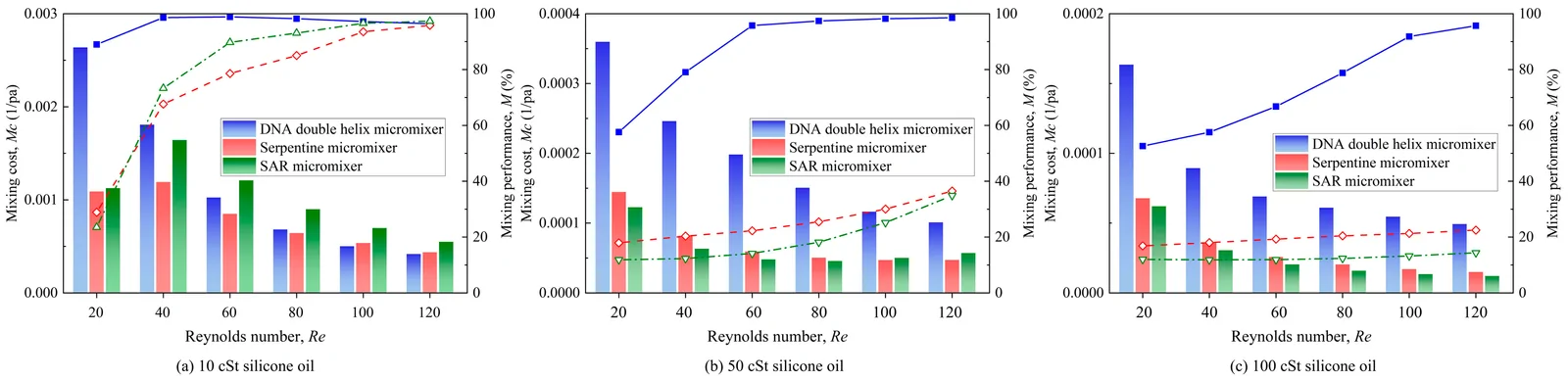
Mixing cost of DNA double-helix micromixer, serpentine micromixer and SAR micromixer for (**a**) 10 cSt, (**b**) 50 cSt and (**c**) 100 cSt silicone oils.

The parameter mixing cost (*MC*) has been employed to further evaluate the overall mixing performance relative to energy consumption, which is defined as the ratio of mixing performance (*M*) to pressure drop (*Pd*). As shown in Figure 18, for 10 cSt silicone oil, the DNA double-helix micromixer exhibits both the highest *MC* and the highest mixing performance among the three designs when *Re* < 60. When *Re* reaches 60, the mixing performance of DNA micromixer is above 98% and is still the highest, whereas the mixing cost of the serpentine and SAR micromixers begins to surpass that of the DNA micromixer. With the further increase in *Re*, the *MC* of the DNA micromixer remains lower than that of the two traditional micromixers.

On the one hand, this is because the chaotic advection in the two-dimensional micromixers becomes progressively stronger as Re increases, resulting in more uniform mixing. In contrast, the DNA micromixer has already achieved nearly completely mixing at *Re* = 60, with a mixing performance above 98%. It means that the chaotic advection is sufficiently strong. Therefore, the further increase in *Re* cannot significantly improve the mixing performance but only increases the pressure drop. On the other hand, the reason behind this can refer to the vortex entrapment effects. Portions of the fluids become trapped in the vortex core, which limits the mass transfer. The mixing performance decreases while the pressure drop continues to increase. As a result, the mixing process in DNA micromixer shows low-cost performance when *Re* exceeds 60.

Moreover, for high-viscosity fluids such as the 50 cSt and 100 cSt silicone oils, both the mixing performance and mixing cost of the DNA micromixer are much higher than those of the serpentine and SAR micromixers. This is because the DNA micromixer could still achieve uniform mixing even for high-viscosity fluids, whereas the two-dimensional designs fail to induce effective chaotic advection under such conditions. Consequently, their mixing performance and mixing cost remain low even at elevated Reynolds numbers.

## 7. Conclusions

The paper proposed a three-dimensional DNA double-helix micromixer enabling efficient mixing of high-viscosity fluids. Fluids flowing through the three-dimensional helix microchannel undergo coupled self-rotation and revolution flows, which generate strong chaotic advection. Owing to the revolution-induced mixing enhancement, rapid and homogeneous mixing can be achieved. For the 10 cSt silicone oil, the DNA micromixer reaches a mixing performance above 98% at *Re* = 30, and the optimum of 99% at *Re* = 50. At higher Reynolds numbers (*Re* ≥ 60), vortex entrapment occurs and slightly reduces the mixing uniformity, but the mixing performance still remains above 95% even at *Re* = 120. For more viscous fluids, such as 50 cSt and 100 cSt silicone oils, the micromixer continues to perform well. It maintains mixing performances above 95% at *Re* = 60 and *Re* = 100, respectively.

In addition, for 10 cSt silicone oil, the DNA micromixer shows a higher mixing cost (*MC*) than serpentine and SAR micromixers when *Re* < 60. For 50 cSt and 100 cSt silicone oils, both the mixing performance and MC are significantly higher than those of the traditional designs. Overall, the DNA double-helix micromixer provides a robust and energy-efficient solution for mixing viscous fluids. Its strong chaotic-advection capability under low-Reynolds-number conditions highlights its potential for practical microfluidic applications involving viscous or multiphase fluids.

## Figures and Tables

**Table 1:** Geometry parameters of the three-dimensional micromixer utilizing DNA double-helix structure.

Geometry Parameters	Value	Dimension
Inlet length (L_0_)	10	mm
Straight channel length (L_1_)	8	mm
Outlet length (L_2_)	8	mm
Base pair length (L_3_)	20	mm
Whole length (L_4_)	90	mm
Microchannel diameter (d)	800	μm

**Table 2:** Fluid characteristics of 10, 50 and 100 cSt silicone oils.

Fluids	Density (kg/m^3^)	Dynamitic Viscosity (mPa·s)
10 cSt oil	9.30 × 10^2^	9.30
50 cSt oil	9.60 × 10^2^	48.0
100 cSt oil	9.65 × 10^2^	96.5

**Table 3:** Inlet velocities of 10, 50 and 100 cSt silicone oils corresponding to *Re* from 1 to 120.

*Re*	Inlet Velocity of 10 cSt Oils (m/s)	Inlet Velocity of 50 cSt Oils (m/s)	Inlet Velocity of 100 cSt Oils (m/s)
1	0.0125	0.0625	0.125
10	0.125	0.625	1.25
20	0.25	1.25	2.5
30	0.375	1.875	3.75
40	0.5	2.5	5
50	0.625	3.125	6.25
60	0.75	3.75	7.5
80	1	5	10
100	1.25	6.25	12.5
120	1.5	7.5	15

**Table 4:** Residence time of 10, 50 and 100 cSt silicone oils corresponding to *Re* from 1 to 120.

*Re*	*t_r_* of 10 cSt Oils (s)	*t_r_* of 50 cSt Oils (s)	*t_r_* of 100 cSt Oils (s)
1	18.7904	3.7581	1.879
10	1.879	0.3758	0.1879
20	0.9395	0.1879	0.094
30	0.6263	0.1253	0.0626
40	0.4698	0.094	0.047
50	0.3758	0.0752	0.0376
60	0.3132	0.0626	0.0313
80	0.2349	0.047	0.0235
100	0.1879	0.0376	0.0188
120	0.1566	0.0313	0.015

**Table 5:** Geometry parameters of the two-dimensional serpentine micromixer and SAR micromixer.

Geometry Parameters	Value	Dimension
Radius (R_s_)	11.08	mm
Splitting length (L_s_)	17.41	mm
Channel width (w)	800	μm
Channel depth (d_s_)	800	μm
Wide splitting channel width (w_1_)	1200	μm
Narrow splitting channel width (w_2_)	800	μm

## Data Availability

The original contributions presented in this study are included in the article. Further inquiries can be directed to the corresponding author.
